# Computational Approach to Fast Analysis of Electrochemical Impedance Spectroscopy

**DOI:** 10.3390/mi17020249

**Published:** 2026-02-15

**Authors:** Cristiano Lo Pò, Stefano Boscarino, Francesco Ruffino

**Affiliations:** 1 Dipartimento di Fisica e Astronomia “Ettore Majorana”, Università di Catania, Via S. Sofia 64, 95123 Catania, Italy; francesco.ruffino@ct.infn.it; 2 Consiglio Nazionale delle Ricerche—Istituto per la Microelettronica e Microsistemi Catania UNIT, Via S. Sofia 64, 95123 Catania, Italy; 3 Research Unit of the University of Catania, National Interuniversity Consortium of Materials Science and Technology (INSTM-UdR of Catania), Via S. Sofia 64, 95125 Catania, Italy

**Keywords:** EIS, gradient descent, fitting algorithm, Powell algorithm

## Abstract

Electrochemical Impedance Spectroscopy (EIS) is a widely used technique for characterizing the electrode–electrolyte interface. EIS analysis can be very complex and tedious. In this work, a fitting algorithm written in *C* is implemented on OriginPro software to avoid the data import/export operation and speed up the analysis. An automated fitting procedure that assigns the initial parameters is implemented for the simplest equivalent circuit. In addition, the possibility of using a custom error function is explored, with results comparable to that of reference software. The developed algorithm is tested on two different case studies.

## 1. Introduction

Electrochemical Impedance Spectroscopy (EIS) is a powerful technique in electrochemistry that is widely used to characterize and qualify an electrode–electrolyte interface [1,2,3,4,5,6,7,8]. The technique consists of applying a small oscillating voltage (typically on the order of 10 mV) in addition to a fixed bias and measuring the resulting oscillating current. The collected data are a set of complex impedance collected at various frequencies, usually equally spaced in logarithmic scale, ranging from kHz to mHz range depending on the characteristic time of the various phenomena occurring on the electrode surface. Data are usually plotted as real impedance vs. imaginary impedance (Nyquist plot) or as |Z| and phase vs. log frequency (Bode plot), and are fitted using some equivalent electrical circuit made up of well-known electrical elements with a specific impedance.

**Figure 1:**
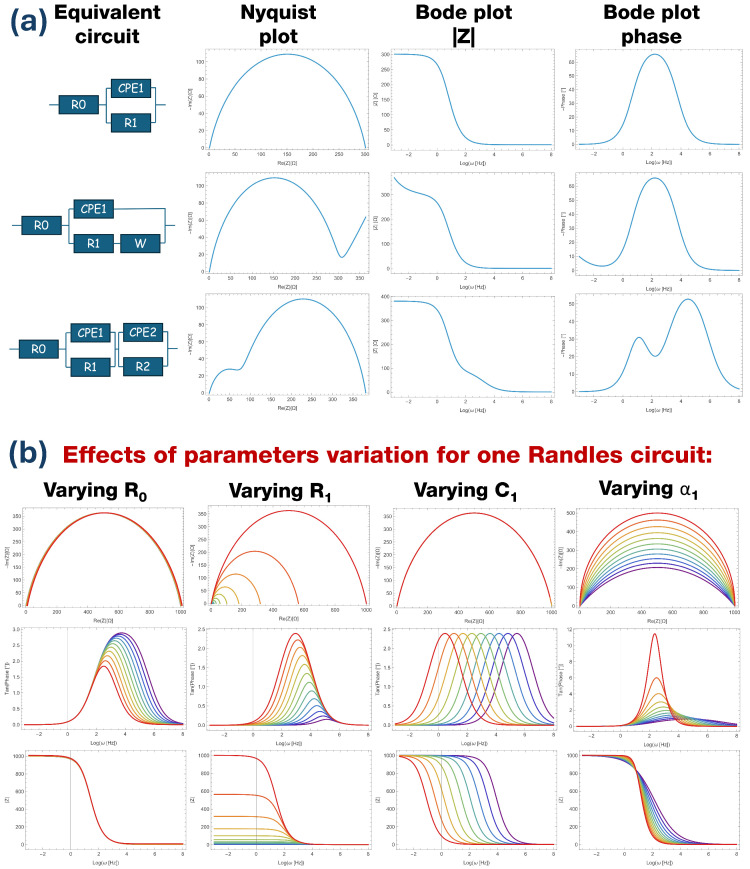
(**a**) Representation of three equivalent circuits (Randles, Randles plus diffusion, double Randles) and their Nyquist and Bode plots. (**b**) Effects of the variation of one parameter (color scale—purple curves: low value; red curves: high value) in a simple Randles circuit on the Nyquist and Bode phase plots.

Some examples of equivalent circuits and their plots are reported in Figure 1a. The Randles circuit [1,2,3,4,5,6,7,8] is made up of a resistance $R_{0}$ in series to a Constant Phase Element (CPE) in parallel with another resistance $R_{1}$, with the following expression for impedance:

(1) $$Z\left(\omega \right)=R_{0}+\frac{1}{{\left(i\omega \right)}^{\alpha_{1}}Q_{1}+1/R_{1}}\;\;\;Z_{\mathrm{CPE}}\left(\omega \right)=\frac{1}{Q{\left(i\omega \right)}^{\alpha }}$$

where $R_{0}$ represents instrumental, electrode, and electrolyte resistance and has values on the order of a few Ω, $R_{1}$ quantifies the charge transfer resistance between the electrode and the electrolyte and depends on the electrochemical reaction occurring at the interface, CPE is a generalized capacitor that takes into account the double-layer capacitance and the ions’ movement that occurs on the electrode surface (where *Q* is called the pseudo-capacitance, measured in Fcm^−2^s^α−1^), and the exponent α is related to the electrode inhomogeneity [9,10,11]. In particular, 0<α≤1; hence, *Q* is not a capacitance. In common experiments, α>0.6 and the Nyquist plot of this circuit is provided by a flattened semicircle; in the Bode plot, the impedance module follows a sigmoidal shape and the phase presents a single peak. When α=1, the CPE becomes a capacitor and the Nyquist plot is a perfect semicircle.

Depending on the electrode polarization, diffusion phenomena take place in the electrolyte and are represented electrically by the so-called Warburg element in series to $R_{1}$. Within this element, the current has $45^{\circ }$ phase with respect to potential, and can be recognized by the characteristic linear behavior at higher resistance in the Nyquist plot and lower frequencies in the Bode plot. The equivalent circuit impedance assumes the following expression:

(2) $$Z\left(\omega \right)=R_{0}+\frac{1}{{\left(i\omega \right)}^{\alpha_{1}}Q_{1}+1/\left(R_{1}+W\left(\frac{1}{\sqrt{\omega }}+\frac{1}{i\sqrt{\omega }}\right)\right)}.$$

A very common experimental situation is present when the substrate is not completely covered by the catalyst or two different catalysts are present on the electrode. In this case, two Randles circuits should be used in series, representing the two contributions to the total impedance:

(3) $$Z\left(\omega \right)=R_{0}+\frac{1}{{\left(i\omega \right)}^{\alpha_{1}}Q_{1}+1/R_{1}}+\frac{1}{{\left(i\omega \right)}^{\alpha_{2}}Q_{2}+1/R_{2}}.$$

Within this configuration, the Nyquist plot assumes the shape of two semicircles, while in the Bode plot the impedance modulus has two steps and the phase has two peaks.

Fitting this kind of data with an equivalent circuit is not a simple procedure. Most commercial data analysis software offers the possibility of fitting a function and carrying out peak deconvolution, but cannot work with complex numbers. Instrument control software comes with the possibility of simple EIS analysis and some specific software is available for free on the web, but these options come with limited functionality and use a very specific data format for input. To avoid the tedious procedure of data export/import along with data format limitations and the usually unpleasant GUI [12,13,14,15,16], a custom fitting algorithm for complex functions has been developed.

A fitting algorithm is just an iterative process that updates a set of parameters inside a function f(x) in order to minimize an error function (also called the cost function of goodness parameter) between experimental points $y_{i}$ and the point computed by $f(x_{i})$ at the same condition $x_{i}$ [17,18].

The simplest goodness parameter is the Residual Sum Squares (RSS), which takes into account the squared distance between the measured data $y_{i}$ and the predicted data evaluated at the same condition $f(x_{i})$. The most used and widely reported goodness parameter is the Reduced Mean Squared Error (RMSE), which depends on the RSS and is divided by the number of points *N*:

(4) $$\mathrm{RSS}=\sum_{i}{\left(y_{i}-f\left(x_{i}\right)\right)}^{2}\;\to \;\mathrm{RMSE}=\sqrt{\frac{\sum_{i}{\left(y_{i}-f\left(x_{i}\right)\right)}^{2}}{N}}=\sqrt{\frac{\mathrm{RSS}}{N}}.$$

Another goodness parameter is the reduced $\chi^{2}$, defined as follows:

(5) $$\chi^{2}=\left(\sum_{i}\frac{{\left(y_{i}-f\left(x_{i}\right)\right)}^{2}}{\sigma_{i}^{2}}\right)\frac{1}{\left(N-M\right)}$$

where $\sigma_{i}$ is the uncertainty on the *i* measurement, *N* is the number of points, and *M* is the number of parameters, making N−M the number of degrees of freedom.

In the case of EIS fitting, the independent variable *x* is the frequency at which every point is collected, while the measured impedance *Z* is a complex number. The squared distance between two points can be generalized to the complex plane by treating the real and imaginary parts separately:

(6) $${\left(a-b\right)}^{2}\to {\left|a-b\right|}^{2}={\left(Re\left(a\right)-Re\left(b\right)\right)}^{2}+{\left(Im\left(a\right)-Im\left(b\right)\right)}^{2}.$$

In this work, a fitting algorithm for EIS data is implemented based on the Powell procedure. It will be shown that a custom error function (or cost function) similar to RSS based on physical considerations and data range leads to faster convergence starting from the same initial parameters within the same number of iterations. Figure 1b shows how Nyquist and Bode plots are affected by variation of the four parameters of a simple Randles circuit (Equation (1)). The Nyquist plot always presents the same semicircular behavior, with strong variations produced only by the decrease of $R_{1}$ or $\alpha_{1}$. The |Z| Bode plot shape is similar to a sigmoid that is not affected by the variation of $R_{0}$ and $R_{1}$, the influence of which instead shifts the plot up and down ($R_{0}$) and affects the step height difference between high and low frequency regimes ($R_{1}$). Capacity variation just produces a frequency shift, while the exponent α changes the slope. The phase Bode plot is strongly affected by any of the parameters; decrease of $R_{0}$ induces a broadening and a shifting of the peak, decrease of $R_{1}$ moves the peak to higher frequencies with less amplitude, $C_{1}$ produces only a shift in resonant frequency, and the exponent α produces a strong peak modification.

Starting from Equation (1), with analytical steps, the phase has the form of a damped Lorentzian (not a Gaussian), as follows:

(7) $$\phi =\frac{\left(R_{1}CSin\left(\frac{\pi \alpha }{2}\right)\right)\omega^{\alpha }}{\left(1+\frac{R_{0}}{R_{1}}\right)+\left(C\left(R_{1}+2R_{0}\right)Cos\left(\frac{\pi \alpha }{2}\right)\right)\omega^{\alpha }+\left(R_{0}R_{1}C^{2}\right)\omega^{2\alpha }}≃\frac{\omega^{\alpha }}{1+\omega^{\alpha }+\omega^{2\alpha }}.$$

Hence, any variation of any parameter affects the phase shape and position. In particular, the maximum phase and the peak frequency are given by:

(8) $$\phi_{max}=\frac{R_{1}CSin\left(\frac{\pi \alpha }{2}\right)}{2{\left(\left(R_{0}R_{1}C^{2}\right)\left(1+\frac{R_{0}}{R_{1}}\right)\right)}^{\frac{1}{2}}+C\left(R_{1}+2R_{0}\right)Cos\left(\frac{\pi \alpha }{2}\right)}$$

(9) $$\omega \left(\phi_{max}\right)=Log_{10}\left({\left(\frac{R_{1}+R_{0}}{R_{0}R_{1}^{2}C^{2}}\right)}^{\frac{\alpha }{2}}\right)$$

For these reasons, an additional term due to the phase difference between experimental and fitted data is included in the modified RSS computation.

The entire code was written and developed in *C* language inside the OriginPro 2021 environment. OriginPro is one of the most widely used software programs for data analysis and plotting in the field of chemistry, material science, physics, biology, and more [19,20]. It offers the possibility of working with a friendly user environment made of tables along with the ability to read any kind of ASCII file organized in columns independently of the original file format. In addition, it is easy to obtain and edit graphs with just a few clicks. For expert users, it can work as an interpreter of codes in various programming languages, with the advantage of reading and writing data directly from tables and updating graphs in real time without issues related to input and output files that use different formatting.

The obtained results are compared using free software [13] that is widely used for EIS analysis. This same software also relies on the Powell algorithm based on the minimization of a reduced $\chi^{2}$ variation:

(10) $$\chi_{software}^{2}=\left(\sum_{i}\frac{{\left(Re\left(Z_{i}^{exp}\right)-Re\left(Z_{i}^{fit}\right)\right)}^{2}+{\left(Im\left(Z_{i}^{exp}\right)-Im\left(Z_{i}^{fit}\right)\right)}^{2}}{Re{\left(Z_{i}^{exp}\right)}^{2}+Im{\left(Z_{i}^{exp}\right)}^{2}}\right)\frac{1}{\left(N-M\right)}$$

which takes into account the number of degrees of freedom. Here, $Z^{exp}$ refers to the experimental complex data and $Z^{fit}$ is the computed impedance.

Moreover, a fully automated procedure is implemented for setting the initial parameters in the case of a simple Randles circuit, opening up the road for a fully automated fitting procedure for the majority of samples. Three case studies are taken into account based on our typical samples and experimental conditions [21,22,23].

## 2. Algorithm Description

**Figure 2:**
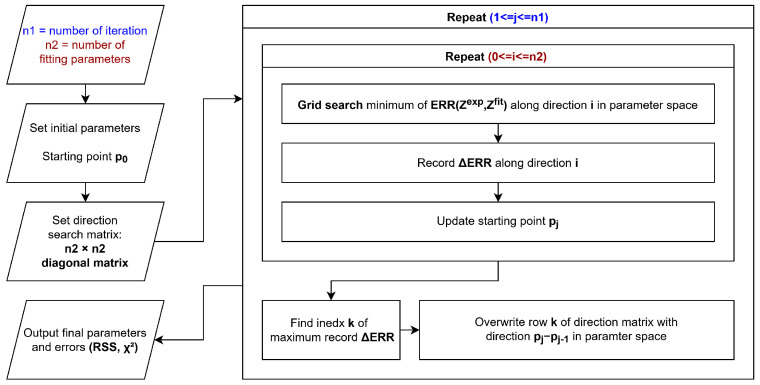
Proposed algorithm schematic.

The algorithm schematized in Figure 2 is a variation of the Powell gradient descent algorithm [24] and consists mainly of two nested iteration cycles.

Initially, the real and imaginary values of the impedance are loaded from the OriginPro worksheet into an array, and the phase and modulus are computed as follows:

(11) $$\phi \left(Z\right)=Tan^{-1}\left(\frac{Im(Z)}{Re(Z)}\right)\frac{180^{\circ }}{\pi }\;\;\left|Z\right|=\sqrt{Re{\left(Z\right)}^{2}+Im{\left(Z\right)}^{2}}.$$

The initial parameters are user-defined as the initial point $p_{0}$ in the parameter space. An identity matrix *u* with the size of the number of parameters is defined, and the rows of this matrix represent directions in the parameter space. The rest of the code is iterated a fixed number of times. For each iteration, a new iterative cycle is performed for the number of parameters. For each sub-iteration, a grid search is performed along every direction defined by the rows of *u* in order to find the minimum of the error function $ERR(Z^{exp},Z^{fit})$. In the grid search, impedance is computed for parameters generated in a fixed number of steps (user-defined) along the selected direction in the parameter space; thus, the custom error function is evaluated from the computed and experimental impedance. When the minimum value of error is reached, the improvement (as $ERR\left(p_{i}\right)-ERR\left(p_{i-1}\right)$) is stored in a ΔERR array and the reached minimum acts as a starting point for the next sub-iteration. After all the directions in matrix *u* have been explored, the row *k* corresponding to the highest element in ΔERR array is replaced with the vector $p_{j}-p_{j-1}$ in parameter space. After a fixed number of iterations, the parameters and the RSS are printed. Additionally, if the ΔERR is below a user-defined threshold, the number of steps in the grid search is increased.

Grid search can appear to be a slow and inefficient method; however, because the code is interpreted and not compiled, the machine precision required for other iterative minimum search approaches is not high enough, making grid search a valid alternative.

The parameters optimized by the algorithm are the resistances $R_{0}$, $R_{1}$, … and the CPE parameters α, $log_{10}\left(Q\right)$. The choice of the log scale for parameter *Q* is related to the fact that it usually spans a range between $10^{-2}$ to $10^{-6}$; within this specific case, grid search is more efficient using the logarithmic scale.

In theory, this can also be used for a series of R-CPE series circuits in the same case, which is useful for modeling the electrode. However, usually EIS data are acquired with fewer than 20 points per decade, and the whole measurement has in general less than 200 total points. The proposed circuit comes with 1+3n fitting parameters (when *n* is the number of R-CPE). With two R-CPE, seven parameters are already required; adding more fitting parameters without increasing the number of points and without significant shapes in the plots (such as peaks in the phase plots or multiple semicircles in the Nyquist plot) will result in overfitting and significance loss.

The code is provided in the Supplementary Materials.

## 3. Test on Real Data

### 3.1. Fitting with One Randles Circuit

**Figure 3:**
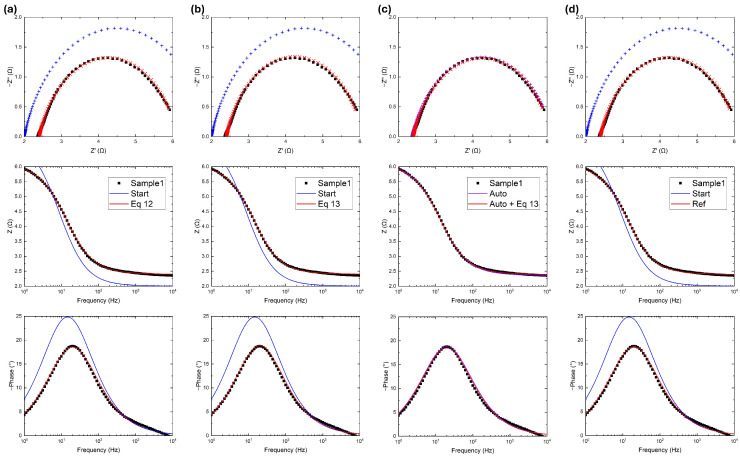
Experimental data (black), initial guess (blue), and fitted data (red) using (**a**) five iterations of the algorithm proposed in this work with the error function in Equation (12); (**b**) five iterations of the algorithm proposed in this work with the error function in Equation (13); (**c**) same as (**b**) except with automatic initial parameter setting; and (**d**) 300 iterations of the reference software. In Bode plots, fitted data are reported with solid curves, while in Nyquist plots are reported as points to visualize the difference from the input data.

A set of experimental data referring to a simple system (measured with an Admiral Squidstat Plus potentiostat; *Admiral Instruments, Tempe, AZ, USA*) is reported in Figure 3. The system in question is voluntarily not reported and described in order to focus on the fitting results. The system can be represented by a Randles circuit with two resistors and a CPE; the initial parameters were chosen to be on the same order of magnitude as the fitted ones, as reported in Table 1. The fitting performance was evaluated using the RSS and $\chi^{2}$, evaluated afterwards as a goodness parameter. In Figure 3a, the algorithm developed in this work uses the RSS as an error function:

(12) $$ERR=\mathrm{RSS}=\sum_{i}{\left|Z_{i}^{exp}-Z_{i}^{fit}\right|}^{2}.$$

The algorithm converges to acceptable values of RSS and $\chi^{2}$ in just five iterations.

In Figure 3b, there are two terms provided by the RSS, as previously defined, plus the RSS defined on the phase difference:

(13) $$ERR=\sum_{i}\left({\left|Z_{i}^{exp}-Z_{i}^{fit}\right|}^{2}+{\left(\phi \left(Z_{i}^{exp}\right)-\phi \left(Z_{i}^{fit}\right)\right)}^{2}\right)=\mathrm{RSS}+\sum_{i}{\left(\phi \left(Z_{i}^{exp}\right)-\phi \left(Z_{i}^{fit}\right)\right)}^{2}$$

Again the algorithm again converges just five iterations, this time to even lower values of RSS and $\chi^{2}$ than in the previous case.

In Figure 3c, the gradient descent operation was performed again with Equation (13), but the initial parameters were automatically assigned as follows, starting from the experimental data. First, $R_{0}$ was assigned as the minimum of the real impedance, and can be identified where the Nyquist plot circle crosses the x-axis near the origin:

(14) $$R_{0}=min\left(Re\left(Z^{exp}\right)\right).$$

The semicircle of the Nyquist plot can provide a valuable guess of the value of $R_{1}$, but depending on the experimental and instrumental conditions it is not always possible to find where the semicircle crosses the x-axis. For this reason, three points were chosen from the experimental array ($Z_{1}=\left\{x_{1},y_{1}\right\}$ at min frequency, $Z_{2}=\left\{x_{2},y_{2}\right\}$ at mid frequency, $Z_{3}=\left\{x_{3},y_{3}\right\}$ at max frequency) and the x-coordinate of the circle was evaluated using the three-point formula:

$$x_{C}=\frac{x_{2}^{2}y_{1}-x_{3}^{2}y_{1}-x_{1}^{2}y_{2}+x_{3}^{2}y_{2}-y_{1}^{2}y_{2}+y_{1}y_{2}^{2}+x_{1}^{2}y_{3}-x_{2}^{2}y_{3}+y_{1}^{2}y_{3}-y_{2}^{2}y_{3}-y_{1}y_{3}^{2}+y_{2}y_{3}^{2}}{2(x_{2}y_{1}-x_{3}y_{1}-x_{1}y_{2}+x_{3}y_{2}+x_{1}y_{3}-x_{2}y_{3})}$$

with $R_{1}$ evaluated as follows:

(15) $$R_{1}=2\left(x_{C}-R_{0}\right).$$

The exponent $\alpha_{1}$ represents the deviation from the ideal capacitor, and in the Nyquist plot is related to the tangent of the semicircle at low frequencies (low impedance) [11]. In this way, the slope on the first n≃10 points is computed and $\alpha_{1}$ is evaluated from the mean slope, as follows:

(16) $$slope_{i}=Tan^{-1}\left(\frac{Im\left(Z_{i+1}^{exp}\right)-Im\left(Z_{i}^{exp}\right)}{Re\left(Z_{i+1}^{exp}\right)-Re\left(Z_{i}^{exp}\right)}\right)\frac{180}{\pi }\;\to \;\alpha_{1}=\frac{mean(slope)}{90}.$$

Using Equation (9) and knowing the other parameters, it is possible to obtain an expression for $Q_{1}$:

(17) $$Q_{1}=\sqrt{\frac{R_{0}+R_{1}}{{\left(2\pi f_{max}\right)}^{2\alpha_{1}}R_{0}R_{1}^{2}}}.$$

Both the automatically settled parameters and the fitted ones have the same order of magnitude as the previous fitted data. The automatic parameter setting feature itself is reliable; after just five iterations, the $\chi^{2}$ value is even lower than in the Figure 3b results, highlighting the advantage of a non-user-dependent procedure.

In Figure 3d, the gradient descent operation is performed by the reference software relying on Equation (10); with 300 iterations, both RSS and $\chi^{2}$ are on the same order of magnitude as with the developed code, another indication of the competitiveness of this work.

### 3.2. Fitting with Two Randles Circuits

**Figure 4:**
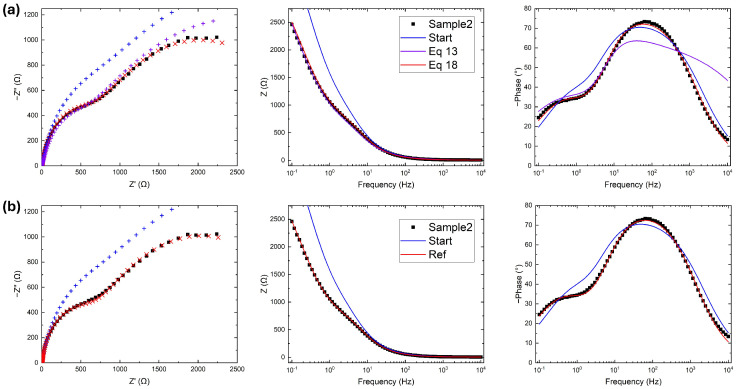
Experimental data (black), initial guess (blue), and fitted data (red) using (**a**) five iterations of the algorithm proposed in this work with the error function in Equation (18) and (**b**) 300 iterations of the reference software. In Bode plots, fitted data are reported with solid curves, while in Nyquist plots are reported as points to visualize the difference from the input data.

A more complex situation measured with an Autolab PGSTAT101 potentiostat; *Metrohm*, *Riverview*, *FL*, *USA* is represented in Figure 4, where two semicircular behaviors are observed in the Nyquist plot and two peaks are clearly visible in the phase Bode plot. In Figure 4a, the fitting operation uses Equation (13), as before; however, the fitted curve in the Nyquist plot is strongly in disagreement with experimental data at both low and high resistances, as reported in Table 2. While the |Z| Bode plot fits perfectly, the phase plot shows the real problem. The two terms of Equation (13) quantify the respective fitting goodness of the two Bode plots. In this specific case study, |Z| spans across three orders of magnitude; consequently:

$${\left|Z_{i}^{exp}-Z_{i}^{fit}\right|}^{2}≃{\left(|Z_{i}^{exp}\left|-\right|Z_{i}^{fit}|\right)}^{2}\gg {\left(\phi \left(Z_{i}^{exp}\right)-\phi \left(Z_{i}^{fit}\right)\right)}^{2}$$

meaning that the modulus has more weight during the gradient descent process at the expense of the phase. To avoid this imbalance, the error function is modified as follows:

(18) $$ERR=\sum_{i}\left(\frac{{\left|Z_{i}^{exp}-Z_{i}^{fit}\right|}^{2}}{{\left|Z_{i}^{exp}\right|}^{2}}+{\left(\phi \left(Z_{i}^{exp}\right)-\phi \left(Z_{i}^{fit}\right)\right)}^{2}\right)$$

with a normalization on the term regarding the modulus. This ultimate error function is close to the one used by the reference software (Equation (10)) with the addition of the phase, and fits the data better, as shown in Figure 4a.

The fitting operation for the reference software is presented in Figure 4b, showing slightly better agreement with the experimental data in the high resistance (low frequency) region. Due to instrumental limitations, this region is noisy; therefore, these discrepancies are not relevant and justify the high values of RSS reported in Table 2. Except for the noise, both RSS and $\chi^{2}$ are again on the same order of magnitude as with the developed code, an indication of the competitiveness and versatility of this work.

### 3.3. Fitting a Randles Circuit with Diffusion

**Figure 5:**
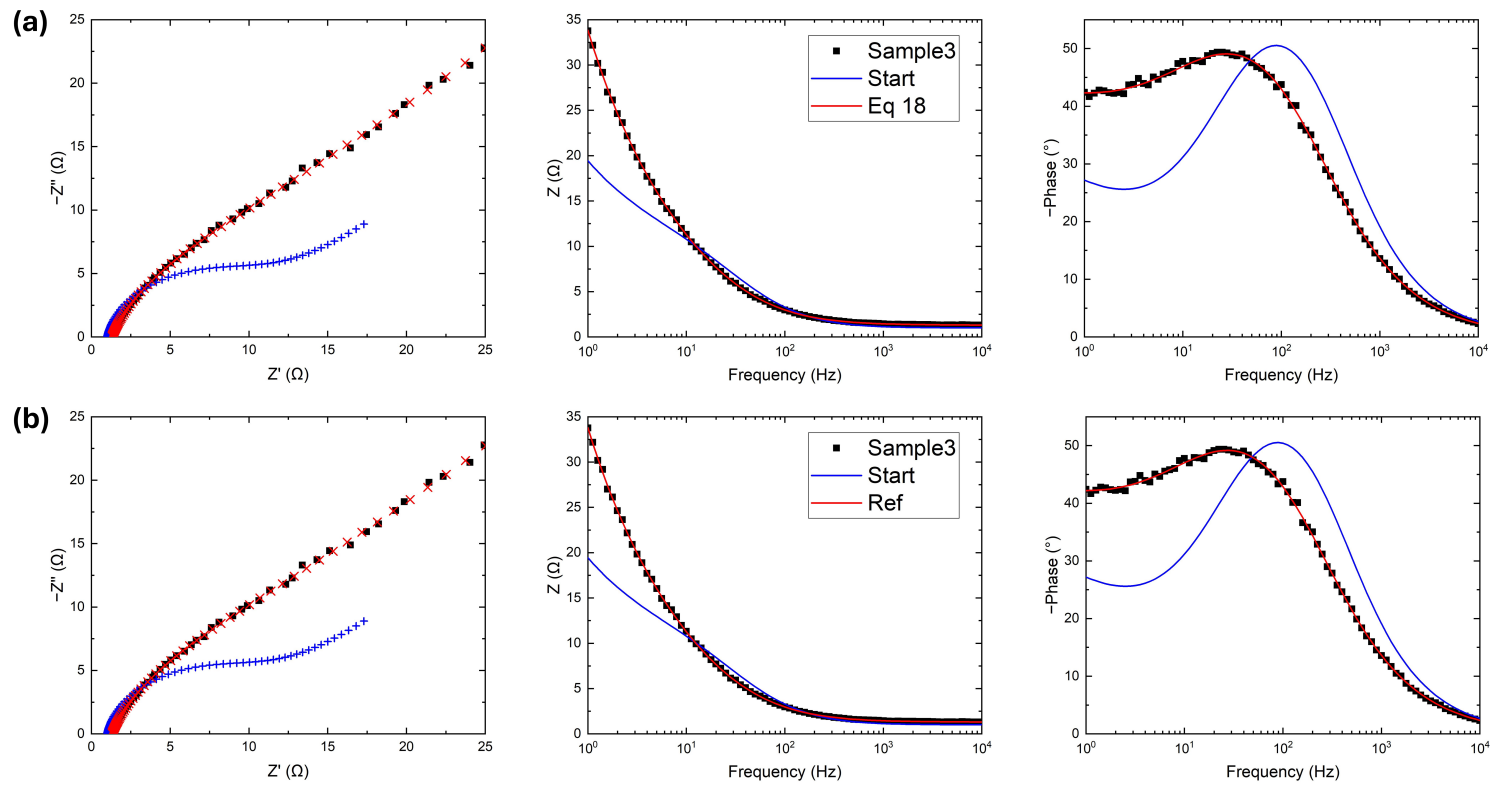
Data (black), initial guess (blue), and fitted data (red) using (**a**) five iterations of the algorithm proposed in this work with the error function in Equation (18) and (**b**): 300 iterations of the reference software. In Bode plots, fitted data are reported with solid curves, while in Nyquist plots are reported as points to visualize the difference from the input data.

A common situation is observed when diffusion takes place and the total impedance becomes that reported in Equation (2). The same procedure described previously is reported in Figure 5, where the data are fitted using Equation (13) as before.

The fitting results reported in Table 3 show very similar values between the proposed algorithm and the reference software; moreover, the final values of RSS and $\chi^{2}$ are the same.

## 4. Conclusions

The first aim of this work is to develop a competitive EIS fitting algorithm running on a data analysis platform to avoid the tedious and time-consuming operation of exporting, importing, and changing the formatting of experimental data files. An additional feature is implemented in terms of automatically fitting the equivalent circuit in the simplest case, which is also the case most treated by our group experimentally.

Furthermore, a study on the error function used for the gradient descent in the fitting algorithm is conducted in order to obtain the best result in just a few iterations. The use of a custom error function is already used in similar specific software for EIS analysis. Because the error function is the fundamental step of every fitting and optimization algorithm, we hope that similar approach can be used and developed in the future to speed up other specific fitting routines, and potentially even machine learning.

## Figures and Tables

**Table 1:** Fitted parameters of Figure 3.

	$R_{0}$	$R_{1}$	$Q_{1}$	$\alpha_{1}$	RSS	$\chi^{2}$
Initial guess	2.00	5.00	0.001	0.8	22.4	1.9
RSS (Equation (12))	2.34	3.73	0.0096	0.79	0.083	$9.6\times 10^{-3}$
RSS + phase (Equation (13))	2.40	3.69	0.0090	0.80	0.076	$9.5\times 10^{-3}$
Auto setting	2.36	3.81	0.0099	0.77	0.16	$1.6\times 10^{-2}$
Auto + Equation (13)	2.39	3.73	0.0099	0.78	0.099	$8.7\times 10^{-3}$
Ref software	2.39	3.72	0.0095	0.79	0.079	$8.3\times 10^{-3}$

**Table 2:** Fitted parameters of Figure 4.

	$R_{0}$	$R_{1}$	$Q_{1}$	$\alpha_{1}$	$R_{2}$	$Q_{2}$	$\alpha_{2}$	RSS	$\chi^{2}$
Initial guess	5.0	600	$8.0\times 10^{-5}$	0.95	4000	$2.0\times 10^{-4}$	0.75	2E7	7.3
Equation (13)	1.0	340	$9.2\times 10^{-5}$	0.99	4460	$3.9\times 10^{-4}$	0.61	1E5	5.7
Equation (18)	5.1	560	$6.0\times 10^{-5}$	0.96	3000	$3.7\times 10^{-4}$	0.74	6E4	$5.5\times 10^{-2}$
Ref software	5.2	570	$5.8\times 10^{-5}$	0.96	2990	$4.0\times 10^{-4}$	0.75	1E4	$2.4\times 10^{-2}$

**Table 3:** Fitted parameters of Figure 5.

	$R_{0}$	$R_{1}$	$Q_{1}$	$\alpha_{1}$	*W*	RSS	$\chi^{2}$
Initial guess	1.0	10.0	$1.0\times 10^{-3}$	0.9	20.0	2E3	5.8
Equation (18)	1.3	15.6	$2.3\times 10^{-3}$	0.81	58.3	1.4	$1.1\times 10^{-3}$
Ref software	1.3	16.2	$2.4\times 10^{-3}$	0.81	57.9	1.4	$1.1\times 10^{-3}$

## Data Availability

Data is contained within the article or supplementary material.

## Supplementary Materials

The following supporting information can be downloaded at: https://www.mdpi.com/article/10.3390/mi17020249/s1, supplementary.rar: archive containing the source code and the raw data file presented in this work.
